# Pharmacological intervention in the field of ototoxicity

**DOI:** 10.1007/s00106-019-0663-1

**Published:** 2019-04-16

**Authors:** G. Laurell

**Affiliations:** Department of Surgical Sciences, Otolaryngology and Head and Neck Surgery, Disciplinary Domain of Medicine and Pharmacy, Faculty of Medicine, Uppsala University, Akademiska sjukhuset ing 78–79, 75185 Uppsala, Sweden

**Keywords:** Cisplatin, Aminoglycosides, Hearing loss, Otoprotection, Inner ear, Cisplatin, Aminoglykoside, Schwerhörigkeit, Otoprotektion, Innenohr

## Abstract

Modern research on ototoxicity goes back to the 1940s, when streptomycin was introduced into clinical practice. Today, aminoglycoside antibiotics and platinum-based chemotherapy, mainly cisplatin, are the most important drugs that damage the inner ear and cause hearing loss. The mode of drug administration as well as drug characteristics influence the likelihood that adequate monitoring of drug pharmacokinetics can be performed. It is not possible to predict the individual risk of treatment with an ototoxic drug, but identification of high-risk treatment protocols is important. There are many studies ongoing with the aim of discovering and developing drugs to treat different types of inner ear disorders. The mechanisms of ototoxicity and subsequent loss of hearing function have been mapped in various experimental models and have provided us with useful information for developing protective treatment. When an ototoxic lesion is established, restoration of hearing function becomes more difficult. For both aminoglycoside antibiotics and cisplatin, a large number of otoprotectors have been suggested. Systemic co-administration of an otoprotector would be the easiest approach to avoid ototoxicity in patients but it may negatively affect the intended pharmacotherapeutic aim of the ototoxic drug. New pharmacological formulations are being developed for local otoprotective treatment. This short review focuses on results from clinical reports on otoprotection in patients treated with aminoglycoside antibiotics and cisplatin. So far there is limited evidence for the safe management of otoprotection in patients. Further high-quality studies are needed to provide reliable data on the safety and effectiveness of pharmacological interventions to reduce drug-induced hearing loss.

## Zusammenfassung

Moderne Forschung zur Ototoxizität geht zurück auf die 1940er-Jahre, als Streptomycin in die klinische Praxis eingeführt wurde. Heute sind Aminoglykosidantibiotika und die platinbasierte Chemotherapie, hauptsächlich Cisplatin, die wichtigsten Medikamente, die das Innenohr schädigen und Schwerhörigkeit verursachen. Die Art der Verabreichung und die Arzneimitteleigenschaften haben Einfluss auf die Wahrscheinlichkeit, dass eine adäquate Überwachung der Pharmakokinetik erfolgen kann. Das individuelle Risiko einer Therapie mit einem ototoxischen Medikament ist nicht vorhersagbar, aber dafür ist die Identifizierung von Hochrisikotherapieschemata umso wichtiger. Es laufen viele Studien mit dem Ziel, Medikamente zur Therapie verschiedener Arten von Innenohrerkrankungen zu finden und zu entwickeln. Die Mechanismen der Ototoxizität und des daraus folgenden Hörverlusts sind in diversen Versuchsmodellen erschlossen worden, daraus ergaben sich nützliche Erkenntnisse für die Entwicklung protektiver Therapien. Ist eine ototoxische Schädigung einmal vorhanden, wird die Wiederherstellung der Hörfunktion schwieriger. Sowohl für Aminoglykosidantibiotika als auch Cisplatin gibt es viele Empfehlungen für otoprotektive Substanzen. Die systemische Mitverabreichung einer otoprotektiv wirkenden Substanz wäre der einfachste Weg, Ototoxizität bei Patienten zu vermeiden, dies kann aber einen negativen Effekt auf das beabsichtigte pharmakotherapeutische Ziel des ototoxischen Medikaments haben. Neue pharmakologische Formulierungen für die lokale otoprotektive Therapie sind in Entwicklung. In dieser kurzen Übersicht liegt der Schwerpunkt auf den Ergebnissen klinischer Berichte zur Otoprotektion bei mit Aminoglykosidantibiotika oder Cisplatin behandelten Patienten. Bisher gibt es nur begrenzt Evidenz für die sichere Anwendung otoprotektiver Substanzen bei Patienten. Weitere qualitativ hochwertige Studien sind erforderlich, um zuverlässige Daten über die Sicherheit und Wirksamkeit pharmakologischer Interventionen zur Verringerung des medikamenteninduzierten Hörverlustes zu liefern.

## Background

The World Health Organization (WHO) has estimated the prevalence of disabling hearing loss to be approximately 5% of the global population. Hearing loss secondary to the use of some ototoxic medications is a well-known, important, and unwanted type of acquired hearing loss. Modern research on ototoxicity goes back to the 1940s, when streptomycin was introduced into clinical practice. Since then, a number of drugs have been considered to be ototoxic, but the majority of them rarely creates a medical problem. Notwithstanding the low risk of ototoxic side effects of some drugs, the physician is expected to be familiar with the undesirable effects and account for risk prediction. When looking for risk factors for ototoxicity, it is obvious that not only the ototoxic potential of the drug itself but also some patient-related and treatment-related factors may influence the risk of drug-induced hearing loss. Among these factors are pre-existing inner ear dysfunction, renal impairment, and concomitant use of ototoxic drugs.

The prevalence of disabling hearing loss is ca. 5% of the global population

The nature of ototoxicity is complex and can be demonstrated as auditory and vestibular toxicity. Clinical findings of drug-induced hearing loss are more frequently reported. To diagnose auditory toxicity, hearing testing should ideally be performed before and after treatment. Today, the use of modern oto-neurological test batteries makes it easier to detect laboratory signs of vestibular toxicity. Aminoglycoside antibiotics and platinum-based chemotherapy, mainly cisplatin, are considered to be the most important drugs that damage the inner ear and cause hearing loss. The ototoxic effects produced by cisplatin treatment limit its clinical use.

The primary objective of the current article is to give a brief overview of the current knowledge on pharmacological intervention in the field of ototoxicity. Ototoxic processes in the inner ear produced by aminoglycoside antibiotics and cisplatin have attracted experimental research interest worldwide. Thus, over the past few decades, our understanding of toxic processes in the inner ear and how these can be prevented at a cellular level has increased tremendously. To date, however, there is still a clear gap between findings in experimental inner ear research and their clinical relevance. Even though many basic findings hold great promise for effective otoprotection, there remains genuine uncertainly about the possibility to transfer them to clinical trials.

## Aminoglycoside antibiotics

Aminoglycoside antibiotics are broad-spectrum antibacterial compounds and are often used for treatment of gram-negative infections. Tobramycin, gentamicin, and amikacin are the most commonly employed aminoglycosides, e.g., in the treatment of pulmonary exacerbations in patients with cystic fibrosis. Adults with cystic fibrosis constitute a group of patients reported to be susceptible to ototoxicity because they require frequent treatments. Another important diagnostic infection group is neonates with sepsis for whom the WHO recommends gentamicin and ampicillin or alternatively penicillin. A systematic review based on six studies of neonates in low- and middle-income countries has estimated an ototoxicity rate of approximately 3% [28]. Nevertheless, the authors conclude that the lack of data on ototoxicity is striking. Aminoglycosides are not only used widely because of their effectiveness but they are also regarded as cheap alternatives when treating severe infections. Some aminoglycoside antibiotics are reported to be more cochleotoxic, whereas others are more vestibulotoxic [21]. There are some important factors identified for aminoglycoside ototoxicity such as higher serum trough levels [4], shorter dosing intervals, greater number of courses, and high cumulative dosage [1]. However, ototoxicity is reported to occur even at a lower number of courses. Most studies on vestibulotoxicity are retrospective. Gentamicin has, e.g., been reported to be associated with no safe serum level for vestibulotoxicity [5]. In a prospective study of patients receiving variable dosing of aminoglycosides, vestibular dysfunction was reported to be more frequent than auditory dysfunction [18].

Some aminoglycosides are more cochleotoxic, others are more vestibulotoxic

Recently, the use of aminoglycoside antibiotics has shifted from multi-daily dosing to once-daily dosing for many approved therapeutic indications. It is often reported that an unrestrained use of aminoglycosides in developing countries may lead to a high number of patients suffering from aminoglycoside-induced hearing loss, although these drugs are given via systemic administration and thereby the treatment should be properly designed and effectuated. However, there are some reports that clinical pharmacology is also advancing in developing countries, which could positively influence the surveillance of hearing function during treatment with aminoglycoside antibiotics.

## The antineoplastic drug cisplatin

Cisplatin is widely used in the treatment of a number of solid malignant tumors. Testicular cancer is particularly sensitive to cisplatin. The drug is also reported to have activity against ovarian, bladder, colorectal, lung, and head and neck cancers. Cisplatin is often used as a part of combined modality treatment for cancer in an advanced stage. In head and neck cancer, cisplatin-based chemotherapy with a palliative intent often leads to initial therapeutic response followed by a gradual resistance. Ototoxicity undoubtedly remains a problem with cisplatin treatment. A number of different dose schedules are used to achieve the optimal antineoplastic effects. The incidence of drug-induced ototoxicity is dose-dependent and related to the single dose level, cumulative dose, and time between treatment courses [35, 36]. However, it has not been proven that the mode of drug administration affects cisplatin ototoxicity [3]. From an auditory point of view, it would be useful to identify the dosing of cisplatin, resulting in the maximum therapeutic outcome with the minimum of toxicity. This might not be possible because the antineoplastic effects are priority number one. Furthermore, it would be extremely complex and would need systematic evaluations of each dose level and diagnosis.

## Similarities and differences

Both aminoglycosides and cisplatin have the potential to be life-saving and show some similarities. The drugs have to be given by the systemic route of administration; they are excreted by the kidneys and may induce nephrotoxicity and produce mainly high-frequency hearing loss. Outer hair cell loss and strial atrophy have been identified in human temporal bones from patients treated with aminoglycosides and cisplatin [12, 23].

Repeated audiograms during treatment can identify high-risk individuals

Besides pharmacological intervention, other otoprotective measures exist to reduce the ototoxic effects. It is likely that audiological monitoring for ototoxicity is of importance especially during high-dose cisplatin treatment. Repeated audiograms during high-dose cisplatin treatment can eventually identify high-risk individuals. However, it is not possible to calculate the exact risk of an ototoxic effect. When severe hearing loss is diagnosed, a simple and useful way to avoid a further ototoxic lesion of cisplatin is dose reduction since a lower dose is associated with a reduced ototoxic effect. Therapeutic drug monitoring can be used to measure drug concentrations to ensure individualized dosing. The mode of drug administration and the drug pharmacodynamics influence the possibility to perform adequate monitoring of drug pharmacokinetics with the aim of evaluating the effects on the inner ear. Plasma concentrations of gentamicin and amikacin are routinely measured in patients and the dose is adjusted when needed to ensure individualized dosing. It has recently been reported in a large study of infants that the impact of gentamicin on the hearing end organ can be minimized by treatments of short durations, monitoring of drug levels, and dose adjustments. It has also been suggested that hearing loss in neonates receiving gentamicin might be attributed to other causes than drug-induced lesions [10]. Free and active cisplatin is more difficult to assess and cisplatin plasma concentration measurements are not used in clinical practice.

Aminoglycoside antibiotics have traditionally been given by repeated daily dosages, while cisplatin is administered less frequently. Studies comparing single-daily dosing and multiple-daily dosing of any aminoglycoside antibiotic are scarce. The results of four studies have been reviewed in a Cochrane database review without any evidence of difference [34]. Theoretically, single-daily dosing may render safer pharmacokinetics with a reduced trough concentration, or even a drug-free period during the treatment course, and thereby less accumulation in the inner ear. Transport of drugs to the inner ear is dependent on the molecular characteristics of the drug. Cisplatin is an extremely reactive molecule that undergoes ligand-exchange reactions with nucleophiles, whereas aminoglycosides are reported to have low protein binding in plasma [17]. Both aminoglycoside antibiotics and cisplatin are water-soluble complexes and are known to poorly penetrate the CNS but are able to pass the blood–labyrinth barriers. The ototoxic mechanisms have been mapped in different experimental settings. Once in the inner ear, these drugs exert their toxic action through several mechanisms, which are out of the scope of this review. In brief, it has been reported that both aminoglycoside antibiotics and cisplatin generate reactive oxygen species in the cochlea and thereby induce mitochondrial dysfunction and induction of apoptotic pathways employing caspase. Even in the cochlea, terminally differentiated cell DNA damage has also been suggested to occur after cisplatin/in connection to cisplatin toxicity.

A series of experiments have demonstrated that kinetics parameters are of importance for cisplatin ototoxicity. The uptake transporters OCT-2 and CTR1 have been shown in mouse models to contribute to the uptake of cisplatin into the cochlea [8, 27]. A delayed elimination of free cisplatin has been found after a single intravenous injection of cisplatin to a guinea pig model [19]. Furthermore, inductively coupled plasma mass spectrometry has been used to quantify the biodistribution of platinum metal in murine and human cochleas. The important finding is that metal platinum is accumulated in the human inner ear long term after treatment, which could indicate that progressive hearing loss seen within several courses is at least partly explained by the influence of persistent formation of a number of inactive platinum complexes in the cochlea [6].

## Otoprotection against aminoglycoside antibiotics and cisplatin

There are many studies in progress with the aim of developing drugs to treat different types of inner ear disorders. The mechanisms of ototoxicity and subsequent loss of hearing function mapped in different types of experimental models have provided us with useful information for developing protective treatment, especially information regarding the molecular mechanisms of apoptotic hair cell death. Pharmacological interventional strategies studied in the laboratory setting in vitro have identified a wide number of substances that can reduce the ototoxic effects of aminoglycoside antibiotics and cisplatin. A limited number of these substances have been studied in vivo, and very few substances have been taken into clinical trials. An otoprotector is a substance that has properties to protect the inner ear against an ototoxic drug. Concomitant administration of an otoprotector is an attractive approach to avoid ototoxicity. When an ototoxic lesion is established, restoration of hearing function will be more difficult. In experimental animals we can test different situations. We can choose between different protection strategies where the most important choices we have to consider are the method of otoprotector delivery, the time window between the administration of ototoxic drugs and the otoprotector, as well as the otoprotector candidates. In experimental studies, otoprotection has been achieved by systemic, intracochlear, intratympanic, and gaseous administration.

There are medical and ethical questions about otoprotection in clinical practice

There are several medical and ethical questions that have to be raised regarding any otoprotection for use in clinical practice. Firstly, it is important that the otoprotection does not cause any drug-to-drug interaction leading to a reduced pharmacological effect of the given pharmacotherapeutic treatment. Secondly, the risk of drug-induced hearing loss of a pharmacotherapeutic treatment must be severe enough to motivate any additional medical measure. Thirdly, the otoprotective measure itself must be without any side effects in order not to cause the patient more problems. Fourthly, otoprotection has to be cost effective.

The lack of a clinical breakthrough generates many questions about the clinical usefulness of otoprotection in severely ill patients. The present short review has the aim of highlighting some earlier findings and will focus on the different modes of otoprotection that have been applied in in vivo research to mitigate drug-induced hearing loss. It is important to clarify that otoprotection during severe infections and cancer treatment of course has significant differences. Given the lack of a standardized view on the otoprotection of aminoglycoside antibiotics and cisplatin, one has to separately look into the possibilities to prevent hearing loss.

Although an extensive amount of experimental research has been performed to emphasize the role of otoprotection against both aminoglycoside antibiotics and cisplatin, there is no generally established clinical guidelines that can be recommended. Thus, one can ask why it has been so difficult to take findings from experimental research to clinical trials even at large institutions. One aspect is that various types of research have been performed with the aim of using ototoxic drugs as experimental models for inner ear disorder and not primarily to find a solution for the problem of drug-induced hearing loss in patients. The main reason for the lack of clinical success is that the human inner ear has unique characteristics that make pharmacological treatment difficult to establish. Thus, there are a number of challenges related to translating preclinical work on otoprotection into clinical trials. One of the most important aspects is that the morphological and physiological construction of the human inner ear differs widely from what is found in experimental animals. For example, there are marked species differences in the arrangement of gap junction proteins in the lateral wall of the cochlea [25]. Moreover, preclinical research is rarely performed on animals with infection or cancer. A sound skepticism is therefore recommended for experimental studies showing substances that have properties to protect the cochlea against ototoxicity. The scarcity of well-designed randomized clinical trials obviously adds to a meager body of clinical knowledge.

As pointed out earlier, there are a lot of data from experimental studies on molecular mechanisms of ototoxicity and otoprotection—the main thing is to know how to use the data in order to avoid hearing loss in patients. Ravi et al. showed more than two decades ago that cisplatin depletes the most important redox system, the glutathione redox system [29]. As both aminoglycoside antibiotics and cisplatin generate reactive oxygen species in the cochlea, drugs that upregulate the antioxidant systems are considered promising candidates for otoprotection, working as a shield for the inner ear and upregulating mitochondria antioxidant machinery. This model is based on the idea that antioxidants interact solely at the intended site of action. One can ask whether this model is correct in a complex in vivo biological system. In order to better understand exposure–response of systemic antioxidants, it would be valuable to identify their precise site of action. Theoretically, the site of action may be located in the systemic circulation, occur at an inner ear level before the intrastrial fluid–blood barrier, or at the target site, i.e., mainly the outer hair cells. There is a long list of antioxidants that have been used in vitro to reduce oxidative stress in the inner ear. This is not amazing as thousands of compounds are reported to have antioxidant capacity.

## Current strategies and possibilities

Otoprotective measures for aminoglycoside antibiotics have most reasonably been based on systemic co-administration. An important issue when developing otoprotection against aminoglycoside antibiotics is the risk of interference with the antimicrobial effects of the latter. A number of studies have shown that systemic administration of antioxidants and scavengers can serve as effective therapeutic interventions to prevent ototoxicity, although very few substances have been tested in an infection context. D‑methionine is a widely used antioxidant and was recently shown not to interfere with the antimicrobial effects of tobramycin [14].

Highly-quality studies of otoprotection in patients treated with aminoglycoside antibiotics are scarce. In clinical trials, aspirin and N-acetylcysteine (NAC) have been studied as otoprotectors [13, 33]. Sha et al. published a 2006 article in the *New England Journal of Medicine* based on a prospective, randomized, double-blind trial featuring 195 patients [33]. They demonstrated a protective effect of aspirin on gentamicin ototoxicity. Review of three randomized clinical studies on co-administration of NAC indicates that this type of otoprotective measure has the potential to be successful. The total number of patients did not exceed 150 [22]. These reports have not been followed by extensive clinical research. The question is why, and it can only be speculated that in an era of increasing antimicrobial resistance, the problem of aminoglycoside antibiotic ototoxicity is no longer considered a significant clinical problem. New aminoglycoside antibiotics are under development that have the potential to be effective without ototoxic side effects.

Otoprotection of cisplatin using concomitant administration of an otoprotector seems to be more complicated, partly because cisplatin is a very reactive molecule and easily reacts with nucleophiles in the blood compartment. Ekborn et al. found that intravenous use of an antioxidant can lower the concentration of free cisplatin in blood, thereby most probably reducing the efficacy of chemotherapy [9]. This finding raises the question about concomitant antioxidant administration in patients and about clinical recommendations. Moreover, Lawenda et al. raised the question about antioxidants administered to cancer patients in general in a review published in 2008 [24]. They concluded that antioxidants may produce tumor protection and therefore continuing research on the use of antioxidants with chemotherapy is warranted. It seems that systemic administration of antioxidants to cancer patients during chemotherapy is still controversial.

Local drug delivery of otoprotectors is an alternative to systemic administration

There may be other ways to attack the problem. An alternative to systemic administration is local drug delivery of an otoprotector. Local drug delivery can be administered as intratympanic injection if the given otoprotector has the chemical characteristics to pass the middle ear–inner ear barriers. Salt and Plontke have systematically evaluated the importance of molecular properties for kinetics after intratympanic administration [31]. They found strong evidence for a selection of suitable candidates for intratympanic administration and that problems related to molecular characteristics make other candidates unsuitable. So, what are the arguments for intratympanic administration of an otoprotector?

This is an attractive approach because cisplatin mainly produces high-frequency hearing loss and the high-frequency tonotopic region is located at the base of the cochlea close to the round window. Another argument for local administration is the lack of drug-to-drug interaction due to the low concentration of the otoprotector in the blood compartment after intratympanic administration [11]. Moreover, high-dose cisplatin treatment is normally given at intervals of 3–4 weeks, making local administration most probably acceptable at these intervals. One problem with intratympanic drug delivery is that a large part of a solution may flow through the Eustachian tube to be swallowed. Premature elimination of a drug injected into the middle ear may be reduced by using a hydrogel formulation [11, 30]. Intratympanic injection of a hydrogel also has disadvantages as it produces temporary conductive hearing loss induced by the intratympanic administration. Besides temporary conductive hearing loss, one should avoid painful injections and also limit the risk of middle ear infection and perforation of the tympanic membrane. A third possibility for otoprotection is inhalation of hydrogen. Since hydrogen gas is considered to have antioxidative properties, a number of researchers have examined the protection of hydrogen in reducing oxidative stress for several medical conditions. Cisplatin-induced hearing loss has been demonstrated in vivo to be limited when using inhalation of hydrogen gas [15]. One drawback is that no attempt has been made to study the effects on tumor cells after intratympanic administration or inhalation.

Regarding cisplatin, there are a number of challenges related to translating preclinical work into clinical trials. High-quality clinical studies are not easy to perform with patients treated with cisplatin, but they are needed to assess the safety, efficacy, and effectiveness of otoprotection in order not to reduce the antineoplastic effects of the drug. One has to point out that many physicians would like to avoid the risk of systemic protective treatment that may be associated with a risk of interference with the antineoplastic effect of cisplatin-based chemotherapy. However, perhaps the gap of knowledge about effects in humans is decreasing. Two randomized clinical trials using intratympanic NAC and dexamethasone, respectively, reported protective effects [26, 32]. Regarding systemic co-administration, some controversy still exists. The use of the cytoprotective agent amifostine is not shown to prevent hearing loss [2]. Thiosulfate seems to be a more promising agent for systemic co-administration, although negative influences on the antineoplastic effect of cisplatin have not been fully assessed. A number of earlier small-scale studies conducted to assess the otoprotective effects of intravenous thiosulfate have yielded promising results [20]. Two recent multicenter studies of pediatric oncologic patients assessing the protective effect of 6 h of delayed intravenous thiosulfate have been reported in high-impact journals [7, 16]. Both studies demonstrated significantly lower ototoxicity in the sodium thiosulfate groups. The results are very promising; however, an increased number of deaths in patients with disseminated disease in one of the thiosulfate-treated groups still leaves some questions about the safety of intravenous thiosulfate for otoprotection [16].

## Practical conclusion

- The most important ototoxic drugs are cisplatin and aminoglycoside antibiotics, i.e., gentamicin, amikacin, and tobramycin.
- Approaches to limit the risk of drug-induced hearing loss can be divided into clinical strategies and pharmacological interventions.
- Clinical strategies involve (1) identifying susceptible patients for ototoxicity (cisplatin), (2) performing drug concentration monitoring (aminoglycoside antibiotics), (3) using serial hearing assessments (cisplatin), and (4) switching to less ototoxic treatment when possible.
- Over the past two decades, an increased body of knowledge has evolved regarding otoprotective mechanisms. Evidence suggests that targeting mitochondrial dysfunction could be a viable way to protect patients’ hearing.
- However, little progress has been made in the management of patients to pharmacologically reduce ototoxicity.
- To overcome the problem with aminoglycoside antibiotics-induced hearing loss, there are reports showing protective effects of co-administration with aspirin or N‑acetylcysteine.
- Delayed intravenous administration of thiosulfate 6 h after terminating cisplatin treatment reduces the ototoxic effect of cisplatin probably without compromising the antineoplastic effects.
- Currently, there are no general guidelines established and more high-quality clinical studies are needed.
